# Allograft for Myeloma: Examining Pieces of the Jigsaw Puzzle

**DOI:** 10.3389/fonc.2017.00287

**Published:** 2017-12-11

**Authors:** Ehsan Malek, Najla El-Jurdi, Nicolaus Kröger, Marcos de Lima

**Affiliations:** ^1^Stem Cell Transplant Program, University Hospitals Seidman Cancer Center, Case Comprehensive Cancer Center, Case Western Reserve University, Cleveland, OH, United States; ^2^Department of Stem Cell Transplantation, University Medical Center Hamburg-Eppendorf, Hamburg, Germany

**Keywords:** allogeneic, hematopoietic stem cells, multiple myeloma, allograft, allograft for myeloma

## Abstract

Multiple myeloma (MM) cure remains elusive despite the availability of newer anti-myeloma agents. Patients with high-risk disease often suffer from early relapse and short survival. Allogeneic hematopoietic cell transplantation (allo-HCT) is an “immune-based” therapy that has the potential to offer long-term remission in a subgroup of patients, at the expense of high rates of transplant-related morbidity and mortality. Donor lymphocyte infusion (DLI) upon disease relapse after allo-HCT is able to generate an anti-myeloma response suggestive of a graft-versus-myeloma effect. Allo-HCT provides a robust platform for additional immune-based therapy upon relapse including DLI and, maintenance with immunomodulatory drugs and immunosuppressive therapy. There have been conflicting findings from randomized prospective trials questioning the role of allo-HCT. However, to this date, allo-HCT remains the only potential curable treatment for MM and its therapeutic role needs to be better defined especially for patients with high-risk disease. This review examines different aspects of this treatment and summarizes ongoing attempts at improving its therapeutic index.

## Introduction

Multiple myeloma is a largely incurable disease that has benefited from the advent of novel agents over the last decade. Allogeneic hematopoietic cell transplantation (allo-HCT) remains the only potentially curative treatment; however, its use is controversial given its high morbidity and mortality. Alloreactive immune effector cells originating from a myeloma-free graft exert graft-versus-myeloma (GVM) effects leading to long-term control of disease (1, 2). Evidence for this GVM effect is provided by reports of sustained molecular remissions achieved by donor lymphocyte infusion (DLI) after allo-HCT (3). Currently, high-dose melphalan followed by autologous hematopoietic cell transplantation (auto-HCT) remains the standard-of-care for suitable patients with MM, as demonstrated by the recent report of the IFM 2009 trial (4). Historically, there have been significant efforts to modulate different elements of allo-HSCT to improve outcomes while minimizing regimen-related toxicity and graft-versus-host disease (GVHD). In the following sections, we will discuss different components of allo-HCT in MM and how they may guide our optimal choice of therapy for MM. There include: patient selection, graft source, conditioning regimen, posttransplant maintenance therapy, and the role of DLI. This review examines these crucial steps of allo-HSCT separately in the context of emerging new data.

## Patient Selection

The risk–benefit ratio of allo-HCT in standard-risk MM is questionable. The BMT CTN 0102 trial randomized standard risk MM patients (defined by Beta-2 microglobluin <4 mg/L, absence of chromosome 13 deletion, and no progression after initial chemotherapy) to tandem auto-HCT (*n* = 366) or auto-HCT followed by matched related donor (MRD) allo-HCT (*n* = 156) (5). There was no difference in relapse rate or overall survival (OS) between the two arms with more treatment-related mortality (TRM) in patients who received auto-HCT followed by allo-HCT (4 versus 11%, *p*-value <0.0001). Similarly, another large study, HOVON-50 trial, did not support the broad application of allo-HCT in MM (6). In this trial, the outcomes of 122 patients who had an HLA-identical sibling donor (donor group) and underwent allo-HCT after a planned auto-HCT were compared prospectively to 138 patients without a donor who underwent maintenance therapy or second auto-HCT after the first auto-HCT. PFS was significantly prolonged in patients who proceeded to allo-HCT compared to maintenance or second auto-HCT. However, after a median follow-up of 77 months complete remission (CR) rate as well as OS was similar between the two groups with higher 6-year TRM in the allo-HCT group (16 versus 3%, *p*-value <0.001). In the European Group for Blood and Marrow Transplantation Non–Myeloablative Allogeneic Stem Cell Transplantation in multiple myeloma (EBMT-NMAM2000 study), patients with an HLA-identical sibling (*n* = 108) receiving auto-HCT/RIC allo-HCT were compared to 249 patients that received tandem auto-HCT. This study demonstrated improved PFS and OS in the auto-HCT/RICallo-HCT arm (OS 49 versus 36% at 96 months, respectively, *p* = 0.030), at the expense of higher TRM (13 and 3%, respectively). Interestingly, the RIC/allo-HCT approach seemed to overcome the poor prognostic implication of del(13) (7). However, none of the patients received any novel agents during induction or maintenance and FISH data was not available for all patients for risk stratification.

Crucial to interpreting these studies is the fact that novel agents and auto-HCT have enhanced OS of MM patients from a median of 3–5 years in the 1990s to over 10 years currently (8). Therefore, it is harder to justify allo-HCT for standard risk disease in the era of novel agents (9, 10).

### Can Allograft Negate High-Risk Factors?

Despite the development of new anti-myeloma therapeutic agents, outcomes for high-risk MM remain dismal. High-risk disease is usually defined by the presence of chromosomal abnormalities including t(4,14), t(14,16), t(14,20), 1q amplification or 1p deletion, 17p deletion and chromosome 13 deletion (i.e., detected on conventional cytogenetic only). This group accounts for 20% of newly diagnosed cases and is associated with early relapse and death (11). A small fraction of MM patients clinically behave aggressively despite lacking “high-risk” chromosomal abnormalities. This includes patients who relapse in a short period of time after auto-HCT (<12 months) (12, 13) or patients diagnosed with plasma cell leukemia (14). Allo-HCT may have an important role to improve outcomes in such patients.

Schilling et al. reported on 101 patients treated with allo-HCT utilizing fludarabine and melphalan conditioning regimen (15). Presence of t(4,14) did not influence outcomes, suggesting that allo-HCT may overcome the influence of this poor prognosis marker. In a multivariate analysis, however, del(13) (HR: 2:34, *p* = 0.03) and del(17) (HR: 2.24, *p* = 0.04) were predictors of relapse. In a report of 143 MM patients who underwent allo-HCT with mostly RIC regimens (77%), the 3-year PFS and OS did not differ for patients with or without del (13), t(4,14), del(17), or t(11,14) (16). In the EBMT-NMAM2000 trial, patients with del(13) allocated to the auto-HCT/allo-HCT group had a survival benefit when compared to the tandem auto-HCT group with PFS and OS of 21 and 47% versus 5% (*p* = 0.026) and 31% (*p* = 0.154), respectively (7). Nishihori et al. reported the outcome of 22 MM patients in very good partial response or CR who underwent HLA-identical sibling allo-HCT as consolidation therapy (17). They showed a 2-year PFS of 74.8%, which compared favorably to 52% PFS in historical controls treated with auto-HCT. In another study by Kröger et al., the 5-year PFS among 16 high-risk MM patients, defined by del (17p) and/or t(4,14), and 57 non-high risk patients who underwent auto-allo tandem was not significantly different (24 versus 30%, *p* = 0.7) (18). In another report of auto-allo among MM patients with chromosome 13 deletion, allo-HCT could overcome del (17) poor prognostic factor [median PFS: 6 months with auto-auto versus not reached with auto/allo SCT (*p* = 0.0002)] (19).

Overall, it seems that allo-HCT may negate some features of high-risk disease. However, there is an unmet need for trials to define the best use of allo-HCT incorporating novel agents for these patients. The ongoing U.S. BMT CNT 1302 trial tests the application of allo-HCT for this population.

## Graft Selection

### Bone Marrow versus Peripheral Blood (PB) Hematopoietic Stem Cells

Mobilized PB hematopoietic cells have been widely adopted as the graft source of choice in allo-HCT for MM since 1994, despite the lack of prospective studies comparing stem cell sources in this setting. The largest retrospective study evaluating graft source was performed by the EBMT registry (20). Transplants performed between 1983 and 1993 (*n* = 334 all with BM source) were compared to transplants done between 1994 and 1998 (BM, *n* = 223 and PB, *n* = 133). Median OS was 10 and 50 months, respectively, for patients transplanted with BM in the earlier and later time periods reflecting an overall reduction in TRM, possibly owing to better supportive care and infection control. The use of PB as the graft source resulted in earlier engraftment without significant OS benefit (20).

### Donor Selection

A matched-related sibling donor is traditionally considered the first choice for patients undergoing allo-HCT. However, a significant proportion of patients will not have such a donor. Kröger et al. conducted a phase II multicenter EBMT trial, where 49 patients with relapsed MM after an autologous HCT received allo-HCT from an unrelated donor (21). One-year TRM rate was 10 versus 53% in patients receiving fully HLA matched versus mismatched grafts. Umbilical cord blood (CB) has also been investigated as an alternative donor source for those without a matched related or unrelated donor. Less stringent matching criteria and lower rates of acute and chronic GVHD are often cited as advantages of using CB. Major disadvantages include delayed neutrophil and platelet engraftment with higher rates of graft failure. Kawamura et al. retrospectively analyzed the Japanese registry data to assess transplant outcomes in 86 patients who underwent a CBT with RIC (RIC-CBT) (22). Six-year PFS and OS were 13 and 15.2%, respectively. Development of chronic GVHD, prior transplantation, and planned tandem autologous HCT followed by RIC-CBT were associated with better OS. The cumulative incidence of 2-year non-relapse mortality (NRM) was 39%, which is higher than recent studies using RIC and allo-HCT for myeloma. Interestingly, NRM was only 6.2% for patients who underwent planned autologous HCT followed by RIC-CBT (22). In another retrospective registry-based study, 95 patients receiving either a single or double CBT were analyzed (23). The 3-year PFS and OS were 24 and 40%, respectively. On subgroup analysis, patient with high-risk cytogenetics had a higher relapse rate, and worse PFS and OS. There are few reports using haploidentical donors; the largest case series included 30 heavy-treated MM patients who underwent haplo-HCT with post-HCT cyclophosphamide as GVHD prophylaxis. The rate of aGVHD, cGVHD, NRM, and relapse rate at 18 months were 29, 7, 10, and 42%, respectively. Median neutrophil and platelet engraftments at day +30 were 87% [95% confidence interval (CI), 66–95%] and 60% [95% CI, 40–75%], respectively. The 18-month PFS and OS were 33 and 63%, respectively. No differences were observed between PB and bone marrow graft in terms of engraftment, GVHD, or PD incidence (24). In another study, 10 patients with relapsed MM who received haploidentical allo-HCT with posttransplant cyclophosphamide; six patients received BM and four patients received PB graft (25). Nine patients engrafted with full donor chimerism; there were no deaths due to acute or chronic GVHD and the 2-year OS was 46%. Given this limited experiences, alternative donor HCT for MM should be investigated in prospective studies before firm recommendations can be made.

### Graft Manipulation

CD34+ selected grafts reduce the incidence of GVHD, potentially compromising the GVM effect with higher posttransplant relapse rates. T-cell depletion in an effective GVHD prophylaxis approach, associated with impaired immune reconstitution and higher incidence of graft failure and posttransplant lymphoproliferative disorders (26). Since RIC allo-HCT relies mainly on alloimmunity and the GVM effect rather than the cytotoxic effect of the conditioning regimen, *in vivo* T-cell depletion using alemtuzumab might not be the optimal approach. Antithymocyte globulin (ATG) may render a direct anti-myeloma effect; therefore, its use for T-cell depletion should be investigated further (27, 28). Soiffer et al. reported a retrospective analysis of patients who underwent allo-HCT for hematological malignancies and showed a lower GVHD incidence with T-cell depleted grafts, but with worse PFS (29). In order to retain the lower TRM associated with RIC regimens and potentiate the GVM effect, some investigators used CD34-selected grafts followed by prophylactic DLI. Smith et al. treated 44 patients with relapsed, high-risk MM with HLA-matched donors with low-dose prophylactic DLI starting 4–6 months posttransplant (30). The reported 2-year PFS and OS were 31 and 54%, respectively. Acute GVHD incidence was 2% and the 1-year TRM rate was 18%. The use of DLI and other posttransplant adoptive immunotherapies to potentiate the GVM effect is discussed below. However, T cell depletion remains controversial and institutional or investigator experience often dictates the approach to graft manipulation.

Shah et al. recently demonstrated the potential of using third-party, *ex vivo* expanded, CB-derived natural killer (NK) cells prior to auto-HCT, potentially providing an anti MM effect in this setting. Importantly, donor NK cells, with an activated phenotype (NKG2D+/NKp30+), were detected *in vivo* in 6 out of 12 (50%) MM patients who were enrolled on the phase I therapy of combined auto-HCT and CB-derived NK cell therapy (31, 32).

The NK antitumor activity is mediated mostly through several receptor families including killer cell immunoglobulin-like receptors (KIRs). KIR loci are located on chromosome 19 and segregated independently from HLA genes; therefore, they are not matched between recipients and donors frequently. KIR mismatch most likely leads to higher NK alloreactivity and several reports showed a significant benefit of KIR mismatch in outcome after allogeneic stem cell transplantation (33, 34).

## Conditioning Regimen

Myeloablative conditioning regimens are generally associated with higher TRM rates mostly due to infection, GVHD, and regimen-related toxicities (35). The most common conditioning regimens incorporate Cyclophosphamide (Cy) and total body irradiation (TBI); Busulfan (Bu) and Cy; Bu/Cy/TBI and Melphalan (Mel) with TBI (Table 1). In the S9321 clinical trial, patients in the allo-HCT arm received the myeloablative conditioning regimen of Melphalan plus TBI. TRM was 53% leading to early closure of the allo-HCT arm (36). In another trial, the Haemato-Oncology Foundation for Adults in the Netherlands (HOVON-24) study, TRM was 30% with dismal PFS and OS for patients who underwent partially T-cell-depleted myeloablative allo-HCT with Cy/TBI conditioning regimen (37). Bensinger et al. compared the outcome of 144 patients who underwent allo-HCT utilizing high-dose myeloablative regimens with 134 patient who received RIC and non-myeloablative (NMA) regimens. Intensity of the conditioning regimen was predictive of higher acute GVHD and TRM rates, earlier relapse, and inferior OS (38). Prior auto-HCT was the main predictor of high TRM after allo-HCT across a variety of conditioning regimen intensities. Other groups reported a similar correlation between prior auto-HCT as well as time between auto-HCT and allo-HCT as another poor prognostic factor and predictor of higher TRM (39). These results suggest that prior exposure to melphalan adds to the toxicity of allo-HCT.

**Table 1:** Outcomes of allogeneic stem cell transplant with myeloablative conditioning regimens in prospective studies.

Conditioning regimen	Number of patients	TRM (%)	PFS/OS^d^ (months)	Reference
CyTBI	334^a^	46	NA/35	(40)
MelTBI				
BuCy				

CyTBI	356^b^	30	NA/55	(20)
MelTBI				
BuCy				

CyTBI	66	24	26/39	(41)
BuCy				

BuCyTBI	80	44	62/39	(35)
BuCy				

Cy/TBI	53	34	18/25	(37)

MelTBI	36	53	NA^c^	(36)

*^a^1983–1993*.

*^b^1994–1998*.

*^c^Trial was stopped due to excessive TRM*.

*^d^At 3 years*.

*TRM, transplant related mortality; PFS, progression-free survival; OS, overall survival; Cy, cyclophosphamide; TBI, total body irradiation; Mel, melphalan; Bu, Busulfan*.

Higher TRM rates associated with myeloablative regimens lead to alternatively exploring NMA and RIC regimens. The CIBMTR database shows a significant shift from intensive regimens from the 1990s to the early 2000s (42). Most of these regimens contain Mel 100–140 mg/m^2^ plus other agents (43). This approach leads to expansion of allo-HCT eligibility criteria by including older patients. Interestingly, higher relapse rates often negated the lower TRM benefit of RIC leading to similar OS.

The strategy of cytoreduction with auto-HCT before RIC allo-HCT has been investigated extensively. Several large prospective randomized trials compared auto-HCT followed by RICallo-HCT to tandem auto-HCT as consolidation therapy (Table 2). However, there is a high degree of heterogeneity among these trials, including use of different conditioning regimens and use of novel agents. Overall, it seems that patients who underwent allo-HCT after auto-HCT are at a higher risk of TRM. A meta-analysis of six studies compared 1,192 patients who underwent tandem auto-HCT to 630 patients who received auto-HCT followed by RIC allo-HCT (44). Recipients of auto-HCT and RIC allo-HCT had higher TRM and CR rates, without PFS or OS advantage.

**Table 2:** Comparison of clinical outcomes of tandem auto-HCT and auto-HCT followed by RIC allo-HCT in prospective randomized trials.

Study	Patients	Graft	Conditioning regimen	GVHD prophylaxis	TRM (%)	CR (%)	PFS (months)	OS (months)
BMT CTN 0102 (5)	Standard risk (84%)	MRD	TBI 2 GY	CSA + MMF	4/11^a^	45/58	46/53	80/77
EBMT/NMAM 2000 (7)	Stable disease	MRD	TBI 2 GY/fludarabine^b^	CSA + MMF	3/13	41/50^c^	12/22^d^	36/49^d^
HOVON-50/54 (6)	All patients	MRD	TBI 2 GY	CSA + MMF	3/16	37/43	22/28^e^	55/55^e^
PETHEMA/GEM2000 (45)	All patients	MRD	Fludarabine/melphalan	CSA + MTX	5/16	11/40	31/–^f^	58/–^f^
IFM99-03/99-04 (46)	High-risk disease^g^	MRD	Busulfan/fludarabine/ATG	CSA + MTX	5/10		35/31	42/35
Bruno et al. (47)	All patients	MRD	TBI 2 Gy	CSA + MMF	2/10^h^	26/55	29/35	54/80

*Gray cells illustrated statistical significance*.

*^a^At 3 years*.

*^b^30 mg/m^2^ × 3 days*.

*^c^At 60 m*.

*^d^At 96 months*.

*^e^At 2 years*.

*^f^Not reached*.

*^g^Beta-2 micro >3 and 13 del at diagnosis with FISH*.

*^h^At 2 years*.

*GVHD, graft versus host disease; TRM, transplant related mortality; CR, complete response; PFS, progression-free survival; OS, overall survival; MRD, matched-related donor; TBI, total body irradiation in Gray (GY); CSA, cyclosporine A; MMF, mycophenolate mofetil; MTX, methotrexate; ATG, antithymocyte globulin*.

### GVHD Prophylaxis

Several investigators have attempted to modify the standard-of-care calcineurin inhibitor-based GVHD prophylaxis regimens. There are preclinical data supporting the use of bortezomib for GVHD prophylaxis. Bortezomib protects against acute GVHD without impairing engraftment or GVT response in murine models of HLA-mismatched allo-HCT (48, 49). This effect is likely due to a suppressive effect of proteasome inhibition on NF-kB, an important modulator of alloreactive T-cell-mediated GVHD (50, 51), in addition to possible modulation effect on antigen-presenting cells (52). Proteasome inhibitors deplete proliferating alloreactive T lymphocytes, suppress TH1 cells, and affect antigen-presenting capacity (51, 52). A phase I/II trial was conducted to investigate the use of bortezomib-based GVHD prophylaxis after HLA-mismatched unrelated donor RIC HCT (53). Bortezomib was given on days +1, +4, and +7 in addition to calcineurin inhibitor and methotrexate with minimal systemic toxicity and GVHD rates similar to HLA-matched allo-HSCT. Koreth et al. investigated the use of bortezomib in addition to tacrolimus and low-dose methotrexate as GVHD prophylaxis in patients with hematologic malignancies that underwent allo-HCT with 1 or 2 HLA-mismatched donor (54). The drug was used in three dose levels of 1, 1.3, and 1.5 mg/m^2^ IV on days +1, +4, and +7 posttransplant (fludarabine and busulfan conditioning regimen). The maximum tolerated dose was 1.3 mg/ m^2^. Cumulative incidence of grade II-IV acute GVHD was 22% with 1-year chronic GVHD incidence of 29% while 2-year TRM rate was 11%.

## Maintenance

Multiple studies showed the benefit of maintenance therapy with lenalidomide or bortezomib after auto-HCT for MM patients (55, 56). In contrast, there are scant data available on maintenance therapy post allo-HCT. Lenalidomide enhances anti-myeloma NK and NK/T-cell response and prolongs PFS and OS when used as maintenance therapy after auto-HCT (55, 57). Lenalidomide augments NK cell-derived anti-myeloma activity while delaying recovery of regulatory T cells posttransplant (58). However, the possible immunostimulatory effect of this drug may lead to rapid onset and higher rates of acute GVHD (59), therefore, complicating its clinical use post allo-HCT. Dosing and timing seems to be crucial for lemalidomide maintenance post allo-HCT affecting NK and T cell activation (58). There is preclinical and clinical studies evidence suggesting that proteasome inhibitors can be safely used post allo-HCT. Moreover, these agents may suppress GVHD while preserving the GVM effect, and this hypothesis is under investigation in the U.S. BMT CTN 1302 trial utilizing the third generation proteasome inhibitor ixazomib as maintenance.

Bortezomib use after allo-HCT may add an anti-myeloma effect (60) without exacerbating GVHD. Kroger et al. administered maintenance bortezomib (Median: 7 months) for 18 MM patients with stable disease after RICallo-HCT (54). The drug was well tolerated with Grade III/IV neuropathy in three patients receiving cyclosporine, while three patients experienced mild aggravation of existing skin GVHD. In another study, 37 MM patients with residual or progressive disease after allo-HCT received bortezomib. Response rate was 73% with two patients experiencing worsening GVHD.

## Donor Lymphocyte Infusion

As discussed previously, most allo-HCT for MM now use RIC regimens at the expense of higher relapse rates. RIC use is especially important in heavily pretreated and/or elderly patients who are not candidates for more intensive conditioning regimens. However, MM is considered to be of only intermediate susceptibility to the graft versus tumor effect. Several strategies have been explored to improve remission rates and decrease relapse risk.

Donor lymphocyte infusion given post allo-HCT is believed to augment the GVM effect. In 1996, Tricot et al. (61) reported CR with a single dose of CD3+ cells in a MM patient who had progressed after allo-HCT. This report was the first “proof of principle” for utilizing DLIs to induce a GVM effect. Although DLIs are mostly given in the context of refractory or progressive disease posttransplant (salvage DLI), this modality of adoptive immunotherapy has also been utilized preemptively posttransplantation (prophylactic DLI) (30, 62–70). Prophylactic DLI given at specific time points or in escalated incremental doses during T-cell reconstitution are believed to enhance donor-derived T-cell reconstitution and to maximize GVM (70) (Tables 3 and 4).

**Table 3:** Summary of studies utilizing donor lymphocyte infusion as a salvage strategy.

Reference	Number of patients	Graft source	T-cell dose ×10^6^ cells/kg (range)	Response rate (%)	GVHD (acute/chronic)	Survival outcomes	Additional treatment
Lokhorst et al. (71)	13	MRD	1–330	62	9/7	54% 1-year OS	

Salama et al. (72)	25	MRD/MUD	2–224	36	13/11	48% 1-year OS	Interferon alpha, before or after DLI

Lokhorst et al. (3)	27	MRD	1–500	52	15/7	Median OS: 18 months	13/27 re-induction chemotherapy before DLI with: VAD, melphalan, or dexamethasone

Lokhorst et al. (73)	54	MRD	1–500	52	31/25	PFS: 19 months	40/54 re-induction chemotherapy before DLI with: VAD, melphalan, or dexamethasone
						OS: 23 months	

El-Cheikh et al. (64)	9	5MRD/4MUD	10–100	75	1/0	50% 2-year PFS	Lenalidomide followed by DLI
						69% 2-year OS	

Montefusco et al. (74)	19	16 MRD/3 MUD	0.5–100 × 10^6^	68	2/5	31% 3-year PFS	Bortezomib/dexamethasone followed by DLI
						73% 3-year OS	

*GVHD, graft versus host disease; MRD, matched related donor; MUD, matched unrelated donor; PFS, progression-free survival; OS, overall survival; DLI, donor lymphocyte infusion; VAD, vincristine, adriamycin, dexamethasone*.

**Table 4:** Summary of studies utilizing prophylactic/pre-emptive DLI post allo-HCT.

Reference	Number of patients	Graft source	T-cell dose ×10^6^ (range)	Response rate (%)	GVHD (acute/chronic)	Survival outcomes	Additional treatment
Alyea et al. (62)	14	MRD	10–30	86	7 total/5 chronic	65% 2-year PFS	Interferon alpha

Badros et al. (63)	14	MRD	120–220	86	10/7	69% 1-year OS	Chemotherapy (DCEP) given post allogeneic transplant fir large tumor burden
							Interferon alpha

Peggs et al. (66)	20	12 MRD/8 MUD	1–100	50	3/2	30% 2-year PFS	
						71% 2-year OS	

Peggs et al. (67)	19	MRD/MUD	1–100	63	9 total	NA	

Kröger et al. (65)	32	11 MRD/21 MUD	0.5–200	78	13/15	54% 5-year PFS	Thalidomide, Bortezomib, or Lenalidomide added if no complete remission achieved with DLI

Smith et al. (30)	44	MRD	0.5–1	NA	1/0	31% 2-year PFS	
						54% 2-year OS	

*GVHD, graft versus host disease; response rate, complete or partial response; MRD, matched related donor; MUD, matched unrelated donor; PFS, progression-free survival; OS, overall survival; DCEP, dexamethasone; cyclophosphamide, etoposide, cisplatin; DLI, donor lymphocyte infusion*.

Response rates as high as 52% have been reported (72, 73). However, in many studies, patients received additional therapies including chemotherapy before DLI to decrease “tumor burden” and improve chances of response. In other reports, DLIs have been combined with either immunomodulatory drugs or proteasome inhibitors.

Graft-versus-host disease is a significant risk associated with DLI that should be taken into consideration before selecting candidates for this treatment modality. The risk of both acute and chronic GVHD is reported to be as high as 60% in some studies. Development of GVHD has been associated with higher response rates but, this has not been consistent across different reports (73). In addition, neither the T-cell dose nor converting patients from partial to full donor chimerism has been consistently associated with higher anti-myeloma response.

A study of frequent chimerism monitoring in 155 patients who underwent allo-HCT for MM showed that two-thirds of relapsing patients had full donor chimerism. Interestingly, one-third of patients had extramedullary disease despite donor hematopoietic reconstitution (75).

Donor-derived cytotoxic T-lymphocytes directed against myeloma-associated antigens such as Wilms tumor 1 (WT1) or other cancer testis antigens (76, 77) may enable a GVM effect without GVHD. Tyler et al. (77) reported a correlation between disease outcomes and WT1 expression by plasma cells in the bone marrow. Development of WT1 cytotoxic T-lymphocytes post allogeneic T-cell depleted transplant and DLI was associated with a GVM effect in the absence of GVHD.

### Chimeric Antigen Receptor (CAR) T Cells

Allogeneic T cells are, as discussed above, frequently associated with off target effects such as GVHD and cytopenias. T cells genetically engineered with CARs expressing receptors to redirected specificity toward tumor cell antigens may generate a very specific GVM response, independent of HLA restriction (78, 79). CAR T cells targeting CD19 showed promising results in recent trials of CD19+ B cell malignancies (80–82) including a few MM cases (83). More recently, advances are being made using CAR T cells against myeloma-specific antigens such as B cell maturation antigen (BCMA), CD138, NY-ESO-1, and kappa-light chain in addition to CD19. Although only a limited number of MM patients have been enrolled on CAR T cell trials, preliminary results are highly encouraging (Table 5). More recently, 33 out of 35 (94%) MM patients with relapsed/refractory disease who enrolled on a phase I trial of CAR T cell BCMA treatment experienced clinical remission within first 2 months of therapy (84).

**Table 5:** Clinical trials using chimeric antigen receptor (CAR) modified T cells in multiple myeloma.^a^

NCT#	Study center	Phase	CAR construct	T cell origin
NCT03070327	Memorial Sloan Kettering Cancer Center, USA	I	EGFRt/B cell maturation antigen (BCMA)-41BBz	Auto/donor
NCT02954445	Southwest Hospital, China	I/II		Auto
NCT01886976	Chinese PLA General Hospital, China	I/II		Auto/donor
NCT02203825	Dana-Farber Cancer Institute, USA	I	Anti-BCMA-CAR-transduced T cells	Auto
NCT02215967	National Cancer Institute, USA	I	CART-138 cells	Auto
NCT02529813	M.D. Anderson Cancer Center, USA	I	D19 + CAR T Cells	Auto
NCT00881920	Baylor College of Medicine, USA	I	Kappa CD28 T cells	Auto

*^a^https://Clinicaltrials.gov accessed on 8/1/2017*.

*Key words: myeloma + CAR T*.

In addition, CAR T cells carry the hope of avoiding allo-HCT altogether. As CAR T cell technology advances, its potential use as a mean to armor “healthy” T cells is emerging as an important consideration. A detailed review of CAR T cells is beyond the scope of this article (85–87).

## Conclusion

Allo-HCT highlights the potential of immune-based cell therapies for MM treatment. Improving risk models incorporating more sensitive minimal residual disease may lead to better patient selection and optimal structuring of different elements of the allo-HCT process in the future. In the U.S., the Centers for Medicare and Medicaid Services is considering MM as an indication for allo-HCT in the setting of an appropriate clinical trial applicable to the Medicare age population. The transplant community will be charged with better defining indications and limitations of this approach. Allo-HCT for MM should continue to be offered within a clinical trial designed to improve and understand the role of this therapeutic modality.

## Author Contributions

Conception and design: EM and ML. Drafting the article: EM and NEJ. Critical revision of the article: ML and NK. Final approval of manuscript and accountable for all aspects of the work: all authors.

## Conflict of Interest Statement

The authors declare that the research was conducted in the absence of any commercial or financial relationships that could be construed as a potential conflict of interest.
